# 

**Published:** 1803-07-01

**Authors:** 


### THE MEDICAL AND PHYSICAL JOURNAL.

CONDUCTED BY

T. BRADLEY, M.D.

R. BATTY, M.D.

AND

A. A. NOEHDEN, M.D.

171620 War 35B

Surg. Gen'l's LIBRARY 5716 Washington, D.C.

VOL. X.

FROM JUNE TO DECEMBER, 1803.

*LONDON:*

PRINTED FOR RICHARD PHILLIPS, NO. 71, ST. PAUL'S CHURCH YARD.

[ William Thorne, Red Lion Court, Fleet Street. ]

### To CORRESPONDENTS.

The Editors will thank Mr. Smith for the description of his improvement of the Trephine; they are in possession of the Drawing.

Mr. Marson's Hints are worthy of the public attention, but the style is too diffusive in its present form.

Dr. Adams's and Mr. Luscombe's Communications are received.

